# Association between Resting Heart Rate and Health-Related Physical Fitness in Brazilian Adolescents

**DOI:** 10.1155/2018/3812197

**Published:** 2018-06-28

**Authors:** Diego Augusto Santos Silva, Tiago Rodrigues de Lima, Mark Stephen Tremblay

**Affiliations:** ^1^Federal University of Santa Catarina Research Center in Kinanthropometry and Human Performance, 88040-900 Florianopolis, SC, Brazil; ^2^Healthy Active Living and Obesity Research Group. Children's Hospital of Eastern Ontario Research Institute, Ottawa, ON, Canada K1H 8L1

## Abstract

The aim of this study was to identify the relationship between health-related physical fitness components (aerobic fitness, muscle strength, flexibility, and body fat) and resting heart rate (RHR) in Brazilian adolescents. The study included 695 schoolchildren (14–19 years) from public schools of the city of São José, Brazil. RHR was evaluated using an automated oscillometric sphygmomanometer. Aerobic fitness was assessed by the modified Canadian Aerobic Fitness Test; muscle strength was measured by handgrip dynamometer; flexibility was assessed by the sit-and-reach test; and body fat was assessed indirectly by sum of two skinfold thicknesses (triceps and subscapular). Sociodemographic variables, habitual physical activity, sexual maturation, and body mass index were the covariates. Cardiorespiratory fitness (*β* = -0.11; 95%CI: -0.14, -0.08) and handgrip strength (*β* = -0.10; 95%CI: -0.18, -0.01) were inversely associated with RHR in boys. For girls, cardiorespiratory fitness (*β* = -0.09; 95%CI: -0.12, -0.06) was inversely associated with RHR. In both sexes, body fat (*β* = 0.50; 95%CI: 0.25, 0.75 for boys; *β* = 0.17; 95%CI: 0.36, 2.72 for girls) was directly associated with RHR. The RHR is measured more easily than the physical fitness tests, so it is recommended to assess adolescent's heath in large surveillance systems.

## 1. Introduction

Heart rate (HR) reflects the number of contractions of the ventricles per unit time and fluctuates substantially with variations in systemic demand for oxygen. Resting heart rate (RHR) monitoring is a simple and noninvasive clinical method related to health prognoses [1]. RHR elevation in adolescents is directly associated with indicators of cardiovascular diseases, such as increased blood pressure levels [2], elevated blood glucose [1], higher total cholesterol concentrations [3], and elevated triglycerides [3].

Considering the evidence regarding the negative health effects associated with elevated RHR in adolescents [1–3], it is relevant to study this issue and to identify factors related to high RHR, such as health-related physical fitness components. It is important to verify the relationship between health-related physical fitness components and RHR, since it is possible to identify modifiable factors in the adolescent population by means of low-cost, easily administered instruments and to propose strategies with the objective of preventing health problems associated with high RHR [1–3].

Most of the findings related to RHR come from investigations aimed at investigating the relationship between RHR and risk factors for cardiovascular diseases, such as blood pressure and inflammatory markers [1, 2]. These risk factors for cardiovascular diseases are influenced by adequate levels of health-related physical fitness components [4, 5]. The literature has reported that adolescents with better performance in physical fitness tests have lower odds of high blood pressure, hypercholesterolemia, and cardiovascular dysfunction [6, 7]. Thus, to investigate the relationship between RHR and health-related physical fitness components will clarify whether the changes in RHR may also be due to the results in physical fitness tests.

The health-related physical fitness components have biologically plausible links to changes in RHR. Higher or improved cardiorespiratory fitness is associated with more efficient myocardial function and lower RHR [8]. Lower results on aerobic fitness tests were associated with lower left ventricular mass, which affects lower resting systolic volume and higher RHR [8]. In addition, higher or improved muscle strength and flexibility levels were associated with neural and muscular adaptations which results in greater parasympathetic nervous system activity reflected in healthy RHR [9, 10]. Excess body fat is associated with the release of inflammatory adipokines into the bloodstream, which are associated with increased sympathetic nervous system activity that results in greater RHR [11]. However, most of this evidence comes from studies with the adult population, which makes it uncertain whether these associations are confirmed with adolescents.

The aim of this study was to investigate the association between health-related physical fitness components (cardiorespiratory fitness, handgrip strength, flexibility, and body composition) with RHR in Brazilian adolescents. Our hypothesis was that better results in health-related physical fitness tests will be associated with lower RHR.

## 2. Materials and Methods

This cross-sectional epidemiological study was carried out in the second half of 2014 in the city of São José, Southern Brazil. The municipality has a Human Development Index (HDI) of 0.809 and Gini index of 0.44 [12].

The sampling process was completed in two stages: being stratified by public high schools (according to density) and clustered by classes considering school shift and grade. To determine the sample size, we followed the procedures suggested by Luiz and Magnanini [13] for a finite population. According to data from the Secretariat of Education of the State of Santa Catarina, we obtained 5,182 students (14–19 years old) who were enrolled in the 2014 school year at 11 eligible public schools of São José and distributed into 170 high school classes (74.8% of students were on the day shift). A confidence level of 1.96 (95% confidence interval), a tolerable error of five percentage points, a prevalence of 50%, and a 1.5 design effect were adopted. We included an additional 20% to compensate for possible losses and refusals and another 20% to control for potential confounders in the association analyses [14]. Under these parameters, the required sample size was 751 students. However, as all students in the selected classes were included, this process resulted in sample larger than estimated, resulting in the collection of data from 1,132 students. Of this amount, 695 students had information regarding RHR and other variables investigated in this study. This sample size had enough statistical power (> 80%) to verify the relationship between RHR and health-related physical fitness components when investigated as continuous variables. In the analyses in which the variables were stratified by sex and treated categorically, the statistical power was less than 80% for the body composition, flexibility, and handgrip strength.

The study was approved by the Ethics Research Committee with Human Beings of the Federal University of Santa Catarina, Brazil. Only subjects who returned the informed consent form signed by parents (<18 years) or by themselves (≥18 years), together with the consent form signed by participants, participated in the study.

Data collection took place in the school environment in the second half of 2014, during the months of August to November. The research team was comprised of undergraduate and graduate students previously familiar with and trained to apply the questionnaire and physical assessments. The questionnaire was answered in the classroom, and data were self-reported by students.

RHR was evaluated using the automated oscillometric sphygmomanometer model Omron HEM 742 (Kyoto, Japan), with validation for adolescents [15]. Participants were informed about the procedures and instructed to remain at rest for at least five minutes, in a quiet and calm environment, with empty bladder, without having exercised for at least 90 minutes before evaluation, not having smoked and not having consumed alcohol, coffee, or tea at least 30 minutes prior to data collection. During RHR collection, participants remained with their backs on a chair, arms flexed at an angle of 90° according to procedures adopted by the American Heart Society [16]. Measurements were performed twice at intervals of fifteen minutes. For this research, the average beats per minute (bpm) rate of the two measurements was used. RHR was measured on the same day of the aerobic fitness test; however, it was performed before the aerobic fitness test. For purpose of analysis, RHR was treated as a continuous measure.

Aerobic fitness was measured using the modified Canadian Aerobic Fitness Test (mCAFT) [17], validated in comparison to indirect calorimetry in Canadian men and women aged 15–69 years [18], and with sufficient discriminatory power to detect high blood pressure levels in young Brazilians [19]. Adolescents had to complete one or more stages of three minutes each (going up and down two steps with increasing intensity) at predetermined rates according to sex and age. The test was terminated when the subject reached 85% of age-predicted maximal heart rate and the end of a 3-minute stage [17], with heart rate measured using a Polar® frequency meter model H7 Bluetooth (Kempele, Finland). In order to estimate VO_2_peak the equation* 17.2 + (1.29 x Oxygen Expenditure) - (0.09 x weight in kg) - (0.18 x age in years) *was used.^17^ In addition, VO_2_peak values were classified according to the distribution tertile for boys (1st tertile ≤ 39.63 mL.kg^−1^.min^−1^, 2nd tertile = 39.64 – 45.20 mL.kg^−1^.min^−1^, and 3rd tertile ≥ 45.21 mL.kg^−1^.min^−1^) and girls (1st tertile ≤ 32.69 mL.kg^−1^.min^−1^; 2nd tertile = 32.70 – 37.56 mL.kg^−1^.min^−1^; and 3rd tertile ≥ 37.57 mL.kg^−1^.min^−1^). We chose this classification because there are no criterion-referenced cut-points for this physical test for the Brazilian population.

Handgrip strength was measured using Saehan® manual dynamometer (Seoul, South Korea). During evaluation, participants remained standing with arms outstretched at the side of the body, with the hand and dynamometer not touching the body. The device was individually sized between the distal phalanges and the palm of the hand, and the adolescent was asked to take maximum inspiration and expiration, followed by a maximal effort to squeeze the dynamometer [17]. The test was performed on both hands alternately, twice, and the best result of each hand was recorded in kilograms (kg) and summed, obtaining the total force. In addition, handgrip strength values were classified according to the distribution tertile for boys (1st tertile ≤ 64.00 kg, 2nd tertile = 64.01 – 79.00 kg, and 3rd tertile ≥ 79.01 kg) and girls (1st tertile ≤ 40.00 kg; 2nd tertile = 40.01 – 49.00 kg; and 3rd tertile ≥ 49.01 kg). We chose this classification because there are no criterion-referenced cut-points for this physical test for the Brazilian population.

Flexibility was measured using the Wells bench for the sit-and-reach test [20]. The test was performed twice and the highest value reached in the test was used [17]. In addition, sit-and-reach test values were classified according to the distribution tertile for boys (1st tertile ≤ 25.19 cm, 2nd tertile = 25.19 – 32.48 cm, and 3rd tertile ≥ 32.49 cm) and girls (1st tertile ≤ 26.16 cm; 2nd tertile = 26.17 – 32.54 cm; and 3rd tertile ≥ 32.55 cm). We chose this classification because there are no criterion-referenced cut-points for this physical test for the Brazilian population.

Body fat was evaluated using the sum of two skinfold thicknesses. The triceps and subscapular skinfold thickness values were collected using the Cescorf® skinfold caliper (Porto Alegre, Brazil), a Brazilian model with design and mechanics similar to the English Harpenden® skinfold caliper (Burgess Hill, United Kingdom), with a presumed constant pressure for any opening of its jaws around 10g/mm^2^, measuring unity of 0.1 mm and contact area (surface) of 90mm^2^. The measurements were taken according to recommendations of the International Society for the Advancement of Kinanthropometry (ISAK) [21]. Anthropometric measurements were performed by a single evaluator with level one ISAK certification. For the present study, the sum of the two skinfolds thickness values was used. In addition, the sum of the two skinfolds thickness values was classified according to the distribution tertile for boys (1st tertile ≤ 16.5 mm, 2nd tertile = 16.6 – 22.3 mm, and 3rd tertile ≥ 22.4 mm) and girls (1st tertile ≤ 26.6 mm; 2nd tertile = 26.7 – 36.9 mm; and 3rd tertile ≥ 37.0 mm). We chose this classification because there are no criterion-referenced cut-points for this physical test for the Brazilian population.

Sociodemographic, physical activity, sexual maturation, and BMI variables were included in the analyses as control variables. Sociodemographic variables were sex (male/female) and age, collected in complete years and later categorized in 14/15, 16/17, and 18/19 years. Physical activity was evaluated by the following question of the Brazilian version of the Youth Risk Behavior Surveillance (YRBSS) questionnaire, used in the United States, translated and validated for Brazil [22]: “During the last seven days, in how many were you physically active for at least 60 minutes a day? (moderate and/or vigorous physical activity).” This question had responses categorized as “inactive/insufficiently active” (zero to four days) and “active” (five days or more) [23].

Sexual maturation was self-assessed by adolescents, using maturational development boards proposed by Marshall and Tanner [24]. These boards contained photographs of the five stages of maturational development, with adolescents being asked to look closely at each photograph and to mark in the questionnaire which photograph most resembled their genital organ size for boys and breast size for girls. Students were individually oriented by same-sex evaluators about the objective and importance of this evaluation. There was a low frequency of adolescents who were in the prepubertal stage (2%), so they were excluded from the analysis. Thus, from this variable, adolescents were classified in “Pubertal” and “Post-pubertal” categories.

Weight and height measures were collected to calculate the BMI. Height was measured with a Sanny® stadiometer with tripod (São Paulo, Brazil) and body mass with G-tech® digital scale (Zhongshan, China). BMI z-scores were computed using age- and sex-specific reference data from World Health Organization [25].

Descriptive and inferential statistical procedures were used, and data normality was verified through mean and median comparison, skewness, kurtosis, and graphs. For the description of continuous variables, mean and standard deviation were used. Student's* t*-test was used to identify differences between variables according to sexes. For categorical variables we used Chi-square test. In order to investigate the relationship between RHR and health-related physical fitness indicators (aerobic fitness, muscular strength, flexibility, and body fat), models were created for each indicator, in which possible associations were analyzed through simple and multiple linear regression, and results were presented as regression coefficients (*β*), 95% confidence intervals (95% CI), and determination coefficients (R^2^). In the adjusted analysis between RHR and models with each health-related physical fitness indicator, sociodemographic variables (sex and age), physical activity, sexual maturation, and BMI were inserted as control variables. Residuals from the final multiple linear regression models were assessed by heteroscedasticity and normality. In addition, the interaction between covariates (age, physical activity, sexual maturation, and BMI) and physical fitness variables was verified. As there was no interaction between variables, analysis of covariance (ANCOVA) was used stratified by sex in order to verify if there were differences in RHR according to the categories (in tertiles) of health-related physical fitness components. In all analyses we calculated the effect size as recommended by the literature [26, 27]. Significance level of p <0.05 was adopted. Statistical analyses were performed using Stata 13.0 software (STATA Corp., College Station, Texas, USA), considering the sample weight and design effect for school.

## 3. Results

A total of 695 students aged 14 to 19 years were included in this analysis. The sample characteristics are shown in Table 1.

In boys, we found that, in the crude linear regression analysis, aerobic fitness and handgrip strength were inversely associated with RHR (Table 2). In the analysis adjusted by age, physical activity, sexual maturation, and body mass index, the aerobic fitness and handgrip strength remained inversely associated with RHR, indicating that the increment of one unit in peak oxygen uptake (mL.kg^−1^.min^−1^) results in a decrease of 0.11 bpm in RHR and that the increase of 1.0 kg results in a decrease of 0.10 bpm in RHR, respectively. In addition, the sum of skinfolds was directly related to the RHR, indicating that the increment of one mm in the sum of skinfolds results in an increase of 0.5 bpm in RHR. These results had a medium effect size (Cohen's f^2^ = 0.27) for the variable aerobic fitness and small (Cohen's f^2^ = 0.03) for handgrip strength and sum of skinfolds (Cohen's f^2^ = 0.05) (Table 2).

In girls, we found that, in the crude linear regression analysis, aerobic fitness and handgrip strength were inversely associated with RHR (Table 2). In the multivariate analysis the aerobic fitness and the body composition were associated with RHR, indicating that the increment of one unit in peak oxygen uptake (mL.kg^−1^.min^−1^) results in a decrease of 0.09 bpm in RHR and that the increase of 1.0 mm in the sum of skinfolds results in an increase of 0.17 bpm in RHR. These results had a small effect size (Cohen's f^2^ = 0.14) for aerobic fitness and small effect size (Cohen's f^2^ = 0.06) for sum of skinfolds (Table 2).

**Figure 1:**
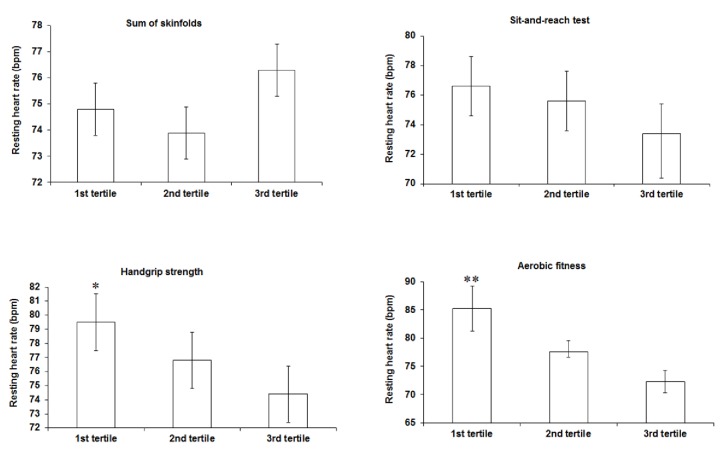
Comparison of resting heart rate (mean and 95% confidence intervals) according to the categories of health-related physical fitness components among males. Analysis of covariance with age, physical activity, and sexual maturation as covariates. *∗* higher values when compared to the third tertile; *∗∗* higher values when compared to the second and third tertile.

In boys (Figure 1) higher RHR was observed in adolescents who had handgrip strength levels classified in the first tertile (lower levels) of the sample distribution when compared to those in the third tertile (higher levels). Higher RHR values were also observed in the adolescents who had VO_2_ peak values classified in the first tertile (lower levels) when compared to those in the second and third tertiles (higher levels).

**Figure 2:**
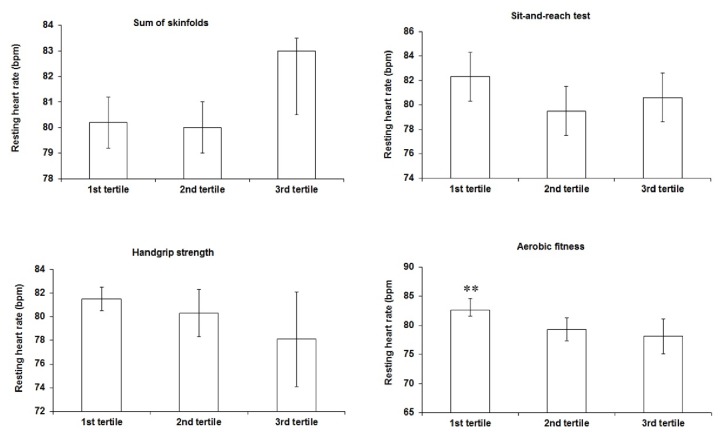
Comparison of resting heart rate (mean and 95% confidence intervals) according to the categories of health-related physical fitness components among females. Analysis of covariance with age, physical activity, and sexual maturation as covariates. *∗∗* higher values when compared to the second and third tertile.

In girls (Figure 2) higher RHR values were observed in the adolescents who had VO_2_peak values classified in the first tertile (lower levels) when compared to those in the second and third tertiles (higher levels).

## 4. Discussion

The main finding of this study was that aerobic fitness was associated with RHR in both sexes, indicating that lower aerobic fitness values were associated with higher RHR values. One explanation for this is that the improvement of cardiorespiratory fitness is associated with increased left ventricular diameter/thickness and increased final systolic volume (due to elevated plasma volume and decreased peripheral resistance), leading to increased stroke volume [8, 28]. The increase in stroke volume leads to a decrease in the number of beats required to maintain cardiac output, decreasing the metabolic load of the heart, contributing to lower RHR [8, 28]. Another explanation is that high levels of aerobic fitness decreases parasympathetic nervous system activity, which reduces the RHR [29]. Other authors found that the improvement of cardiorespiratory fitness in adolescence reflects better cardiovascular health indicators, such as healthy blood pressure levels, favorable lipid profile, and lower risk of morbidity and mortality in adult life [30, 31]. In this way, promoting the improvement of the cardiorespiratory fitness of children and adolescents is to promote healthier people throughout life.

For boys, we also found inverse association between handgrip strength and RHR values. A cohort study followed 38,000 men for 30 years (from adolescence to adulthood) and found that men with high muscle strength in adolescence had a decreased risk of later cardiovascular disease events (HR: 0.88; 95% CI: 0.77-0.99). However, low muscle strength was associated with increased risk of cardiovascular disease mortality during middle age (HR: 1.31; 95% CI: 1.02-1.67) [32]. As the RHR is an indicator of cardiovascular health [2], we can hypothesize that adequate levels of muscle strength in men can be an indicator of adequate levels of RHR. The biological plausibility of the association between muscle strength and RHR is the fact that the development of muscular strength is directly related to the intensity and duration of the counterresistance effort [33]. The increase in the cardiometabolic demands concomitant with the counterresistance effort (necessary to improve muscle strength) leads to an imbalance between the tonic activity of the sympathetic accelerator neurons and parasympathetic depressors in favor of the greater vagal domain, resulting in lower RHR [8, 33, 34].

For girls an association between handgrip strength and RHR was not found. In the crude analysis we found an inverse association between these variables, but when the model was adjusted, especially with the inclusion of BMI, this association was not maintained. A systematic review reported there are few studies with women that aimed to identify the association between muscular strength and cardiovascular health indicators [35]. These few studies have reported a significant association between muscle strength and indicators of body fat or metabolic syndrome [35]. Research completed with Australian adolescents found an association between handgrip strength and cardiovascular indicators; however, when the BMI was included in the analysis model this association disappeared [36]. This Australian study did not stratify the sample by sex. Rioux et al. [37] aimed to investigate the association between handgrip strength and cardiometabolic health in a large Canadian sample of children and youth. The authors found that for girls high handgrip strength was significantly associated with lower odds of cardiometabolic risk factors independent of age, BMI z-score, and cardiorespiratory fitness. For boys handgrip strength was not associated with lower odds of cardiometabolic risk factors [37]. Thus, there are inconsistent findings in the literature. Based on a recent systematic review, our study is timely, as the authors of this review concluded that there is a lack of comprehensive understanding of factors, such as sex, that could impact cardiometabolic risk factors in youth and children [38]. We also emphasize that as there are few studies that aimed to associate muscle strength with RHR in adolescents, we cannot establish whether our results agree or disagree with the literature. Possible justification for nonassociation between muscle strength and RHR in girls would be related to lower absolute strength values in girls compared to boys, which results in lower compression of blood vessels by the muscle during contraction, which may result in reduced adaptations related to cardiac hypertrophy and reduction in RHR [39]. In addition, the lack of association between muscle strength and RHR in girls may also have occurred because of the hormonal changes that affect adolescents. The hormonal changes in girls due to puberty result in lower performances in strength tests, which may have made the girls sample homogeneous in relation to handgrip strength, making it difficult to find differences between the groups investigated [33, 40].

As we found in the present study, other studies [3, 41] have identified a direct relationship between increased body fat and increased RHR. Some hypotheses indicate that an increase in body fat results in increased sympathetic nervous system activity [11, 42]. In addition, adipose tissue secretes angiotensinogen, which provokes angiotensin II formation and further activation of sympathetic nervous system activity. This increased activity of the sympathetic nervous system results in greater RHR [11, 42]. In addition, it is possible that the association between body fat and RHR may have been mediated by the level of physical activity and/or aerobic fitness, considering that both factors are related to body fat and RHR [1, 7].

We found no association between flexibility and RHR. Although it is a topic that needs further research, the literature reports that adequate levels of flexibility have beneficial effects on cardiovascular health, such as blood pressure and heart rate variability [9]. Recent evidence in adults suggests that improving the flexibility of a single muscle group or different muscle groups may reduce blood pressure and RHR [9, 43]. One potential mechanism for this situation may be related to the improvements in sympathetic control of vasomotor tone which can be caused by improving muscle flexibility [44]. Indeed, it has been suggested that the decrease in sympathovagal balance can be associated with increased trunk flexibility [45]. Therefore, any notable improvement in RHR with improved flexibility may be related to a decrease in vasomotor tone (i.e., reduced vagal inhibition) [9]. The literature did not investigate these effects in adolescents and so we cannot say that this would be expected in them. Proportional differences in the length of the upper and lower limbs of schoolchildren may have contributed to the absence of the association between flexibility and RHR, since such asymmetry may imply underestimation or overestimation of the results and affect the validity of the test used [46].

For all results of this study, the effect size was small or medium, reflecting little meaningful interpretations in a practical sense [26, 27]. In other words, what we want to report is that these results should be interpreted with caution. In boys, cardiorespiratory fitness and handgrip strength will need to increase 9.0 mL.kg^−1^.min^−1^ and 10 kg, respectively, to result in a decrease of one bpm in RHR. For body fat in boys it will need to decrease 2.0 mm in sum of skinfolds to result in a decrease of one bpm in RHR. In girls, cardiorespiratory fitness will need to increase 10.0 mL.kg^−1^.min^−1^ to result in a decrease of one bpm in RHR. Body fat in girls will need to decrease 5.0 mm in sum of skinfolds to result in a decrease of one bpm in RHR. The most important result of the present study is that associations were found between health-related physical fitness components an d RHR. This demonstrates that acting on the improvement of these components in the pediatric population may be useful in preventing cardiovascular complications in adolescence.

The use of RHR as a prognosis for health problems has been evidenced in the literature [1–3, 19, 41]. However, some previous studies did not consider in the analyses the possible confounding effect of aspects directly related to RHR, such as indicators of physical fitness or levels of physical activity [1, 2, 41]. In the present study, we considered these variables in the statistical analyses and found an inverse association between RHR and aerobic fitness (boys and girls) and muscular strength (boys) and direct association with body fat (boys and girls). Such associations had a weak magnitude of association. However, the results found reinforce the use of RHR as a useful measure to be used in adolescents and can be used in large monitoring systems because they are easier to apply than physical fitness tests.

This study has some notable limitations. The cross-sectional design of this study may be considered a limitation, since it prevents the establishment of causality and temporality among variables. The low sampling power for the comparisons between the tertiles of the health-related physical fitness components and RHR is another limitation of the research. Physical fitness tests depend on the adolescent's motivation to perform it, and, for this reason, it can be a measure with limitations. Another limitation was that although schoolchildren had their RHR values measured after five minutes of absolute rest (and were told not to exercise for at least 90 minutes prior to assessment), it is possible that some of those evaluated may have performed moderate to vigorous intensity physical activity prior to 90 minutes from the assessment of RHR, which may have implied a possible bias of the verified results. This occurs because physiological alterations resulting from activities of moderate to vigorous intensity may imply RHR values above the basal value for prolonged periods [47]. However, a strength of this study was its methodological rigor in the training of the team and the use of direct physical measures using validated instruments for data collection, providing greater assurance of the accuracy of the findings.

In conclusion, the present study demonstrated that cardiorespiratory fitness and handgrip strength were inversely associated with RHR in boys. For girls, cardiorespiratory fitness was inversely associated with RHR. In both sexes body fat was directly associated with RHR. These associations were independent of age, physical activity level, stage of sexual maturation, and BMI of adolescents. RHR measurement is a viable alternative for use in large health monitoring systems because they are easier to employ than physical fitness tests.

## Figures and Tables

**Table 1:** Characteristics of the sample.

	**Male **	**Female **		
	**(n = 337)**	**(n= 358)**		
	**Mean (SD)**	**Mean (SD)**	**p**	**Cohen's D**
**RHR (bpm)**	76.1 (13.6)	81.1 (10.9)	<0.01*∗*	0.40
**Age (years)**	16.2 (0.1)	16.0 (0.1)	<0.01*∗*	0.18
**Weight (kg)**	65.7 (0.5)	58.5 (0.5)	<0.01*∗*	0.60
**Height (cm)**	172.7 (0.3)	161.3 (0.2)	<0.01*∗*	1.69
**BMI (kg/m** ^**2**^ **)**	22.0 (3.6)	22.4 (4.0)	0.04*∗*	0.11
**Triceps skinfold (mm)**	10.8 (5.1)	18.7 (6.9)	<0.01*∗*	1.30
**Subscapular skinfold (mm)**	10.8 (4.8)	15.5 (7.2)	<0.01*∗*	0.76
**Sum of skinfolds (mm)**	21.5 (9.5)	34.3 (13.5)	<0.01*∗*	1.09
**Aerobic fitness (mL.kg** ^-**1**^ **.min** ^-**1**^ **)**	42.7 (5.4)	35.3 (3.7)	<0.01*∗*	1.60
**Handgrip strength (kg)**	71.8 (17.3)	45.4 (10.4)	<0.01*∗*	1.85
**Sit-and-reach test (cm)**	28.7 (7.6)	29.5 (7.6)	0.08	0.11

	**n (**%**) **	**n (**%**)**	**p**	**Cramer's V**

**Physical activity **				
Active	99 (57.9)	72 (42.1)	<0.01*∗*	0.01
Less active	238 (45.4)	286 (54.6)		
**Sexual Maturation**				
Pubescent	244 (52.7)	219 (47.3)	<0.01*∗*	<0.01
Post-Pubescent	93 (40.1)	139 (59.9)		

RHR: resting heart rate; SD: Standard Deviation; *∗*p<0.05; Cohen's D: effect size for comparison between groups; Cramer's V: effect size for chi-square test.

**Table 2:** Relationship between resting heart rate and health-related physical fitness components in adolescents.

**Variable**		**Crude Analysis**				**Adjusted Analysis** ^b^			**Cohen's *f*** ^***2***^
	**ß** ^**a**^	** (95%CI)**	***p***	**R** ^**2**^	**ß** ^**a**^	** (95**%**CI)**	***p***	**R** ^**2**^	**(df = 5)**
**Male**									
Aerobic fitness	-0.09	(-0.11; -0.07)	<0.01*∗*	0.15	-0.11	(-0.14; -0.08)	<0.01*∗*	0.21	0.27
Handgrip strength	-0.13	(-0.20; -0.06)	<0.01*∗*	0.02	-0.10	(-0.18; -0.01)	0.28*∗*	0.03	0.03
Sit-and-reach test	-0.15	(-0.31; 0.15)	0.07	0.01	-0.15	(-0.34; 0.03)	0.10	0.03	0.03
Sum of skinfolds	0.11	(-0.22; 0.24)	0.10	0.01	0.50	(0.25; 0.75)	<0.01*∗*	0.05	0.05
**Female**									
Aerobic fitness	-0.05	(-0.08;-0.03)	<0.01*∗*	0.04	-0.09	(-0.12; -0.06)	<0.01*∗*	0.12	0.14
Handgrip strength	-0.10	(-0.20; -0.01)	0.02*∗*	0.01	-0.04	(-0.15; 0.05)	0.36	0.04	0.04
Sit-and-reach test	-0.04	(-0.17; 0.08)	0.47	0.01	-0.05	(-0.19; 0.09)	0.48	0.03	0.03
Sum of skinfolds	-0.05	(-0.12; 0.01)	0.14	0.01	0.17	(0.36; 2.72)	<0.01*∗*	0.06	0.06

a: regression coefficient; b: adjusted analysis by sex, age, physical activity, sexual maturation, and body mass index; R^2^: determination coefficient; CI: confidence interval; Cohen's f^2^:effect size for multiple linear regression; df: degree freedom.

## Data Availability

All article data can be accessed by contacting the corresponding author.
